# Quantitative assessment of German Holstein dairy cattle colostrum and impact of thermal treatment on quality of colostrum viscosity and immunoglobulins

**DOI:** 10.1186/s13104-020-05019-z

**Published:** 2020-03-30

**Authors:** Abdulwahed Ahmed Hassan, Sebastian Ganz, Florian Schneider, Axel Wehrend, Izhar U. H. Khan, Klaus Failing, Michael Bülte, Amir Abdulmawjood

**Affiliations:** 1 Institute of Veterinary Food Science, Justus-Liebig-University Gießen, Frankfurter Street 92, 35392 Giessen, Germany; 2 Klinik für Geburtshilfe, Gynäkologie und Andrologie der Groß- und Kleintiere mit Tierärztlicher Ambulanz, Justus-Liebig-University Gießen, Frankfurter Str. 106, 35392 Giessen, Germany; 3 Department of Veterinary Public Health (DVPH), College of Veterinary Medicine, Mosul University, Mosul, Iraq; 4 Agriculture and Agri-Food Canada, Ottawa Research and Development Centre, Ottawa, ON K1A 0C6 Canada; 5 Biomathematik und Datenverarbeitung, Justus-Liebig-University Gießen, Frankfurter Str. 95, 35392 Giessen, Germany; 6 Institute of Food Quality and Food Safety, Research Center for Emerging Infections and Zoonoses (RIZ), University of Veterinary Medicine Hannover, Bünteweg 17, 30559 Hannover, Germany

**Keywords:** Holstein dairy cattle colostrum, Color, Fat, Dynamic viscosity, Colostrometer, Digital %Brix refractometer, Thermal treatment

## Abstract

**Objective:**

This study aimed to determine the color, fat, viscosity, IgG concentration, %Brix and refractive index of fresh postpartum colostrum of German Holstein dairy cattle and assess the impact of different thermal treatments on the visual and dynamic viscosity, in association to IgG concentration, of colostrum that can be used for pasteurization process.

**Results:**

Of the total 40 fresh postpartum colostrum, the color of colostrum (ranging from white-pale yellow to yellow and dark-yellowish), fat (1.4–8.2 100 g^−1^), IgG (4–116 mg mL^−1^), %Brix (8.5–35.4%), refractive index (1.3454–1.3905 nD), visual (ranging from watery to liquid and thick) and dynamic (4.9–219 cp) viscosity, were recorded. Statistical analysis between visual and dynamic viscosity of fresh colostrum showed significant correlation coefficients (*r*_s_ = 634). Moreover, a significant correlation between viscosity and three IgG concentrations was also observed. Heat-treated colostrum showed dynamic viscosity ranged from 25 to 3066 cP, where dynamic viscosity of colostrum before- and after heat-treatment showed no significant correlation. Treated colostrum at 60 °C/60 min and 63.5 °C/30 min containing IgG concentration ≤ 80 mg mL^−1^ and ≤ 68 mg mL^−1^ showed no significant change in the viscosity and can successfully be applied for pasteurization of first postpartum colostrum.

## Introduction

Colostrum contains essential and non-essential bioactive compounds [e.g., immunoglobulins (Igs)] and nutrients for passive transfer of immunity to newborn calves [1–3]. After parturition, newborn calves need to ingest 3–4 L of high-quality colostrum containing ≥ 50 mg mL^−1^ IgG within the first 6 h after birth to protect them from pathogens until their own immune system is developed [1, 4]. Therefore, estimating the quality of first-milking colostrum on farms by measuring the specific gravity or IgG concentration prior to feeding calves is a useful management tool to improve calf health [5–7]. The present study was designed to evaluate color, fat, and IgG (mg mL^−1^, %Brix and refractive index) concentration as well as visual and dynamic viscosity of first postpartum colostrum of German Holstein dairy cattle. Previous studies have applied heat treatment and pasteurization of colostrum, at various temperature and time, either under laboratory conditions using water bath or directly on the farm using a commercial batch pasteurization system [5, 8, 9]. For establishing an optimal heat process that can minimally impact on viscosity and immunoglobulins, different thermal treatments (temperature and time) were investigated in this study.

## Main text

### Methodology

#### Physico-chemical analysis

Initially, 16 German Holstein dairy cattle farms, located in Hessen, Germany, were selected for collection of 40 colostrum samples. For this study, the first colostrum, from dairy cattle between three and seven years age, was collected within 30 to 60 min after parturition. All samples were collected between March 2017 to Oct. 2018 with mean monthly (ranged from 3.2 °C to 18.8 °C) and seasonal temperature (ranged from 1.1 °C to 20 °C) (Additional file 1: Table S1). Each sample was visually analyzed for color and viscosity. Dynamic viscosity was measured with native colostrum program (1 rpm at 30 °C/60 s.) using a viscometer DV3T touchscreen rheometer (AMETEK Brookfield, Germany), and two programs (1.0 and 0.1 rpm at 30 °C/60 s) used for samples treated at 63.5 °C/30 min. The fat content was determined using the Gerber method [10].

#### Immunoglobulins

Colostrum IgG (mg mL^−1^) was measured by a colostrometer [11, 12], with an optimum 37 °C temperature (Pfizer Animal Health GmbH, Germany). A digital handheld refractometer DR201-95 (A. Krüss Optronic, Germany) was used to determine the %Brix and refractive index (nD) [13]. The Brix refractometer scale of ≥ 20% (50 mg mL^−1^) IgG corresponds to a good as compared to ≤ 20% Brix (≤ 50 mg mL^−1^) IgG corresponds to a low quality colostrum.

#### Thermal treatments

Three thermal treatments (60 °C/60 min, 63.5 °C/30 min and 72.0 °C/15 s) were performed in a water bath to determine the cut-point temperature that may impact on the viscosity in relation to the IgG concentration [8, 14]. The water bath was calibrated and adjusted to ± 0.5 °C with an additional digital thermometer (Testo 112 Type NTC, Germany) and thermometer type UT330A (Reichelt Elektronik, Germany).

#### Statistical analysis

The statistical data analysis was performed using BMDP v8.1.0 and StatXact v9.0.0 statistical software packages [15, 16]. The values are described as arithmetic mean, standard error of mean (**± **SEM) and standard deviation (SD), p = 0.05 deemed as significant. For analyzing the relationship between fat content, visual and dynamic viscosity and IgG (mg mL^−1^, %Brix and nD) concentration, Pearson correlation coefficient (*r*) and rank correlation coefficient analyses were performed for ordinal-scaled data in accordance with Spearman’s rank-order correlation coefficient (*r*_s_) and logarithmic methods.

### Results

#### Color and fat content

The color of 37 samples ranged from white-pale yellow to yellow and dark-yellowish. The other three samples collected from healthy dairy cattle showed a slightly pinkish due to hemorrhage per diapedesis. The fat concentration of colostrum samples ranged from 1.4 to 8.8 100 g^−1^ (mean 5.4 100 g^−1^) (Table 1). There was no significant correlation observed between the fat and color gradation as well as between fat and IgG (mg mL^−1^, %Brix and nD) concentrations (Table 2).

**Table 1:** Analysis of fat and IgG [concentration, %Brix, refractive index (nD)], and dynamic viscosity of fresh and heat-treated dairy cattle colostrum samples

Parameter	Mean (average)	± SD	± SEM^a^	Minimum	Maximum
Fat (100 g^−1^)	5.41	2.03	0.32	1.4	8.8
IgG (mg mL^−1^)	57.65	32.71	5.17	4.0	116.0
%Brix	20.32	6.12	0.96	8.5	35.4
nD	1.3640	0.010	0.001	1.3454	1.3905
Dynamic viscosity (cP)^b^	34.55	41.27	6.52	4.9	219.0
Dynamic viscosity (cP)^c^	658.87	911.09	145.89	5.2	3.066

^a^SEM: standard error of mean

^b^First postpartum colostrum

^c^Heat-treated colostrum (63.5 °C for 30 min)

**Table 2:** Correlation co-efficient analysis of dairy cattle colostrum samples using various physico-chemical parameters

Parameter	IgG concentration			Viscosity of first colostrum	
	mg mL^−1^	%Brix	nD	Visual	Dynamic^§^
Fat %	*r* = 0.242 *p* = 0.133	*r* = 0.099 *p* = 0.543	*r* = 0.081 *p* = 0.619	*r*_s_= -203 *p* = 0.210	*r* = 0.237 *p* = 0.140
mg mL^−1^	–	*r *= 0.894 *p* = 0.001	*r *= 0.887 *p* = 0.001	*r*_s_= 0.896 *p* = 0.001	*r *= 0.575 *p* = 0.001
%Brix		–	*r *= 0.991 *p* = 0.001	*r*_s_= 0.841 *p* = 0.001	*r* = 0.742 *p* = 0.001
nD			–	*r*_s_= 0.839 *p* = 0.001	*r* = 0.772 *p* = 0.001
Visual viscosity of first colostrum				–	*r*_s_= 0.634 *p* = 0.001

^§^Logarithmic values; *p* value: < 0.05 considered significant; *r*: correlation coefficient; *r*_s_: Spearman’s rank-order correlation

#### Immunoglobulins

**Fig. 1:**
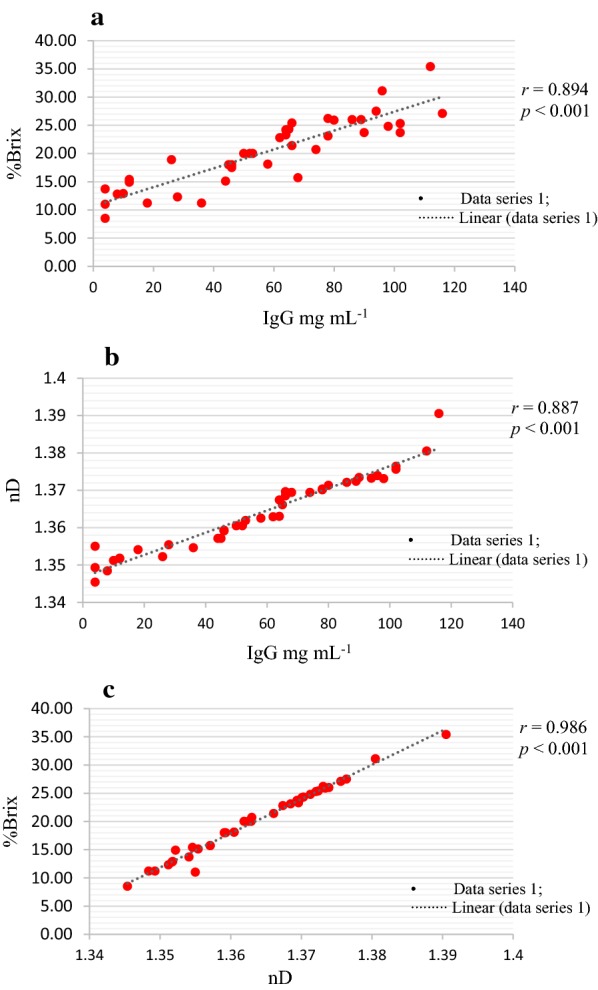
Statistical correlation analysis of fresh dairy cattle colostrum samples between IgG mg mL^−1^ and %Brix (**a**); IgG mg mL^−1^ and nD (**b**); and  %Brix and nD (**c**)

The IgG (mg mL^−1^) concentration of fresh colostrum samples using a colostrometer ranged from 4.0 to 116 mg mL^−1^ (Additional file 1: Table S1). Based on the cut-point (≤ 50 mg mL^−1^) concentration, 15 (37.5%) and 25 (62.5%) samples were classified as low and high quality colostrum, respectively. Similarly, the %Brix of IgG ranged from 8.5 to 35.4%Brix, where based on cut-point (≤ 20% Brix) value, 17 (42.5%) and 23 (57.5%) samples were classified as low and high quality colostrum, respectively. Furthermore, the refractive index of IgG values ranged from 1.3454 to 1.3905 nD with a similar percentage (42.5 and 57.5%) of samples, based on the cut-point (≤ 1.3596) value, were classified as low and high quality colostrum (Table 1). The relationship congruency between IgG mg mL^−1^ and %Brix values revealed that 38 (95%) samples were in agreement with the colostrometer and refractometer methods. The statistical relationship revealed significant coefficient correlation between IgG mg mL^−1^ and %Brix (*r *= 0.894), IgG and nD (*r *= 0.887) and %Brix and nD (*r *= 0.991) (Table 2), while linear regression correlations are illustrated in Fig. 1 (panels a–c).

#### Visual and dynamic viscosity

The visual viscosity of first fresh colostrum was categorized as watery (n = 14; 35%), liquidy (n = 17; 42.5%) and thick (n = 9; 22.5%) (Additional file 1: Tables S1, Additional file 2: Table S2). The 17 samples containing high-fat (7.0–8.8 100 g^−1^) were classified into: watery (n = 9; 53%), liquidy (n = 6; 35%) and thick (n = 2; 12%). Although the statistical analyses revealed no significant correlation between fat and visual viscosity, a significant correlation between visual viscosity and IgG (mg mL^−1^, %Brix and nD) concentration was recorded (Table 2; Additional file 3: Fig. S1A–C).

The dynamic viscosity of first colostrum ranged from 4.9 to 219 centipoise (cP) (Table 1), where similar to visual viscosity, significant correlation was found between dynamic viscosity and IgG (mg mL^−1^, %Brix and IgG nD) concentration but not with the fat content (Table 2; Additional file 3: Fig. S1D–F). Statistical analysis results, using Spearman’s rank-order correlation method, showed significant correlation coefficients (*r*_s_ = 634; *p = *0.001) between visual and dynamic viscosity for all colostrum samples (Table 2; Additional file 3: Fig. S1G).

#### Analysis of thermally treated colostrum

After treating colostrum at 60 °C/60 min, all 14 watery and 15 liquid samples, the viscosity was changed to liquid, while the two liquid and nine thick samples showed slight to moderate coagulation, where ≤ 80 mg mL^−1^ pasteurization cut-point IgG concentration was recorded. The viscosity of watery colostrum samples treated at 63.5 °C/30 min changed to liquid, while the liquid and thick samples formed slight to moderate coagulation with ≤ 68 mg mL^−1^ cut-point IgG concentration. However, 17 samples treated at 72.0 °C/15 s showed moderate coagulation (cut point: ≤ 45 mg mL^−1^) as compared to the remaining 23 samples that changed into solid due to excessive coagulation (Additional file 2: Table S2).

After heat-treatment of all colostrum samples at 63.5 °C/30 min, only 28 samples, measured at program 1.0 rpm at 30 °C/60 s, showed dynamic viscosity ranging from 25 to 310 cP. The remaining 12 samples, tested at program 0.1 rpm at 30 °C/60 s, showed dynamic viscosity ranging from 1.407 to 3066 cP (Table 1). There was no significant correlation observed between dynamic viscosity of fresh and heat-treated (63.5 °C/30 min) samples (Table 2).

### Discussion

In this project, we have studied vital physico-chemical properties and their correlation in first fresh postpartum colostrum samples collected from healthy German Holstein dairy cattle. Furthermore, this study evaluated the relationship between the impact of heat-treatment on the colostrum viscosity and threshold of IgG concentrations. Generally, physical properties of colostrum such as color gradation and visual viscosity provides an initial impression of the quality status of colostrum. In this study, a correlation between color gradation and degree of viscosity was assessed where a significant (*p* = 0.05) relationship was observed. Gross et al. [17] reported that colostrum has a wide range of color spectra ranging from pale-white to dark-brown/red compared to the dairy cow milk color. The color gradation of colostrum increased progressively from pale to dark with more fat, protein and IgG as well as dietary composition also considered as contributory factors [17–19]. However, a correlation between color and viscosity was not previously reported. Furthermore, the fat content varied in contrast to Quigley et al. [20] where a higher fat content (9.2–31.6 100 g^−1^; mean: 23.6 100 g^−1^) was reported. On the other hand, Kehoe et al. [21] measured the average colostrum fat 6.7 100 g^−1^ as compared to 3.6 100 g^−1^ [22]. In contrary to the previous study [17], the present study results showed no significant relationship between color gradation and fat as well as between fat and various degrees of viscosity. This weak correlation with these parameters can possibly be due to the color variation as compared to the viscosity and fat concentration.

For assessing IgG concentration, previous studies have recommended on-farm tools to provide precise and reproducible results [6, 13, 20, 23–27]. Based on these studies, we used colostrometer and refractomter as alternative indirect, rapid and accurate tools to assess the quality of German Holstein dairy cattle colostrum by estimating the IgG concentration. Both methods showed a high degree (≥ 95%) of similarity in the classification of colostrum IgG concentration where strong correlation coefficients (0.894 and 0.887) with %Brix and nD were observed. Interestingly, these results are higher than previously reported results [24, 26] where the correlation coefficients of IgG and %Brix were relatively low (0.71 and 0.75). However, our results were similar to Morrill et al. [27] with a correlation coefficient of 0.86. The colostrum quality, in the present study, revealed disparate individual IgG concentrations leading to a high variation but lower (69.9 mg mL^−1^) than the previously reported IgG concentration [20]. The IgG concentration data obtained from colostrometer (mg mL^−1^) and refractomter (%Brix and nD), in our study, revealed that 37.5%, 42.5% and 42.5% samples did not show congurence to the recommended IgG cut-points (≥ 50 mg mL^−1^; ≥ 20%Brix and ≥ 1.3596 nD). On the other hand, 72.9 mg mL^−1^ IgG mean concentration was determined in Jersey dairy cattle colostrum ranging from 12.8 to 154.3 mg mL^−1^, and 32.8% of samples had < 50 mg mL^−1^ IgG concentration with a mean (21.24%) %Brix of fresh colostrum, %Brix values ranging from 10.5 to 28.6% with recommended breed-specific ≥ 18% cut-point [27]. In congruence with our  %Brix results, conventional (62.5%) and organic (56.1%) Danish dairy cattle colostrum samples had equal or exceeded 22%Brix cut-point, with significant variation ranging from 8.3 to 35.1% [7]. Our cut-point level of good quality colostrum was determined at ≥ 20%Brix corresponded to ≥ 50 mg mL^−1^ IgG concentration using colostrometer. Chigerwe et al. [13] and Bielmann et al. [25] suggested 22%Brix as an optimal cut-point level as compared to the recommended levels (18%, 21% and 23%) for Jersey dairy cattle colostrum [6, 9, 14, 24–27].

Heat-treatment of colostrum, either in experimental conditions or commercial batch pasteurization system, was previously investigated to determine the efficiency of pasteurization on viability of microorganisms to reduce calf exposure to bacterial pathogens, change in viscosity (degree of coagulation) and degradation of IgG [5, 8, 9]. The present study indicates that pasteurizing colostrum at 60 °C/60 min containing ≤ 80 mg mL^−1^ IgG concentration have a minimal impact on the viscosity, whilst pasteurizing colostrum at 63.5 °C/30 min containing ≤ 68 mg mL^−1^ IgG concentration have a moderate impact on the viscosity. Our study results correspond to the previous study where pasteurizing colostrum at 63.5 °C/30 min using a commercial batch pasteurizer produced a mildly thick coagulation viscosity compared to 72 °C/15 s where heat-treatment caused a solid form of colostrum especially in samples containing IgG concentration > 50 mg mL^−1^ [5]. Furthermore, viscosity or IgG concentration remained unaltered when colostrum was treated at 60 °C for 120 min using the Rapid Visco Analyzer (RVA). However, high quality colostrum containing ≥ 73.0 mg mL^−1^ IgG concentration had a significant impact on IgG concentration and viscosity at 63 °C as compared to colostrum containing < 73.0 mg mL^−1^ IgG concentration [28]. Similarly, no change in IgG concentration was observed when colostrum was treated at 60 °C for 60 min using a commercial on-farm batch pasteurizer [29]. Interestingly, similar to our findings, colostrum treated at various temperatures (57, 60 and 63 °C) and time (30, 60 and 90 min) did not affect viscosity [8]. Donahue et al. [30] reported first-milking colostrum containing IgG between 97.4 - 36.4 mg mL^−1^ treated at 60 °C for 60 min did not show a negative impact on IgG concentration. Interestingly, change in the viscosity of colostrum containing IgG ≥ 80 mg mL^−1^ has not been reported yet. Similarly, pasteurization of first colostrum of buffaloes and cows was carried out at 63 °C for 30 min, 60 °C for 60 min and 72 °C for 15 s where no effect on the IgG concentration and viscosity of colostrum was observed at 60 °C for 60 min compared to a study where no impact on IgG concentration and quality of colostrum treated at 60 °C and 63 °C for 30 and 60 min was observed [13, 31]. However, the cut-point of pasteurization, in our study, at different heat-treatment maintained the viscosity, IgG concentration and high quality of colostrum.

### Conclusion

Analysis of quantitative IgG concentration of first colostrum using colostrometer or refrectometer method is useful prior to initiating on-farm pasteurization step using commercial colostrum pasteurizers. Pasteurization of first colostrum containing IgG ≤ 80 and ≤ 68 mg mL^−1^ at 60 °C/60 min and 63.5 °C/30 min did not significantly impact on visual and dynamic viscosity and quality of colostrum. Therefore, these termal treatments can successfully be applied commercially for pasteurizing colostrum. Moreover, the treatments, subsequently, allow to adjust the volume of colostrum, without altering properties, for feeding and successful transfer of passive immunity to the calves.

## Limitation

In this study, IgG-ELISA test on colostrum samples before and after different heat-treatments and their correlation were not performed due to limited sources of funding.

## Supplementary information

**Additional file 1: Table S1.** Colostrum and environmental data including sampling date, IgG concentration, viscosity and temperature collected at the time of sample collection.

**Additional file 2: Table S2.** Categorization of IgG concentrations (mg mL^−1^) and visual viscosity of fresh first postpartum and heat-treated colostrum samples.

**Additional file 3: Fig. S1.** Statistical correlation analysis of dairy cattle colostrum samples, Panels A, B and C: Visual viscosity with IgG (mg mL^−1^, %Brix and nD) concentrations. Panels D, E and F: Dynamic viscosity with IgG (mg mL^−1^, %Brix and nD) concentrations. Panel G: statistical correlation analysis between visual and dynamic viscosity.

## Data Availability

The dataset used in the study can be provided by the corresponding author upon request

## Abbreviations

Igs: Immunoglobulins
IgG: Immunoglobulin G
nD: Refractive index
cP: Centipoise
(*r*): Pearson correlation coefficient
(*r*_*s*_): Spearman’s rank-order correlation
LHL: Landesbetrieb Hessisches Landeslabor
