# Supplementary material for: Quantitative assessment of German Holstein dairy cattle colostrum and impact of thermal treatment on quality of colostrum viscosity and immunoglobulins
Source: BMC Res Notes. 2020 Mar 30;13:191. doi: 10.1186/s13104-020-05019-z (PMC7106573; doi:10.1186/s13104-020-05019-z)
Supplement: Supplementary file 1 — Additional file 1: Table S1. Colostrum and environmental data including sampling date, IgG concentration, viscosity and temperature collected at the time of sample collection. [file 13104_2020_5019_MOESM1_ESM.docx]

**Supplementary Table S1:** Colostrum and environmental data including sampling date, temperature, viscosity and IgG concentration collected at the time of sample collection.

| Sample  code | Farm code | Date of collection | Monthly mean temperature  (ºC)* | Seasonal mean temperature (ºC)* | Optical viscosity | IgG  (mg mL^-1^) |
| --- | --- | --- | --- | --- | --- | --- |
| 01 | A-1 | 28.04.2017 | 7.9 | Winter: 1.1  Spring: 10.1  Summer: 18.3  Autumn: 9.8 | liquid | 52 |
| 02 | B-2 | 03.05.2017 | 14.5 |  | Thick | 112 |
| 03 | C-3 | 07.05.2017 |  |  | Liquid | 68 |
| 04 | D-4 | 08.05.2017 |  |  | Watery | 28 |
| 05 | E-5 | 26.05.2017 |  |  | Thick | 96 |
| 06 | F-6 | 19.06.2017 | 18.3 |  | Liquid | 62 |
| 07 | G-7 | 01.07.2017 | 18.8 |  | Liquid | 46 |
| 08 | F-6 | 03.07.2017 |  |  | Thick | 86 |
| 09 | H-8 | 04.07.2017 |  |  | Liquid | 50 |
| 10 | I-9 | 05.07.2017 |  |  | Watery | 12 |
| 11 | J-10 | 06.07.2017 |  |  | Liquid | 64 |
| 12 | F-6 | 07.07.2017 |  |  | Liquid | 64 |
| 13 | F-6 | 07.07.2017 |  |  | Liquid | 66 |
| 14 | I-9 | 07.07.2017 |  |  | Liquid | 79 |
| 15 | F-6 | 11.07.2017 |  |  | Thick | 90 |
| 16 | F-6 | 13.07.2017 |  |  | Liquid | 80 |
| 17 | K-11 | 15.07.2017 |  |  | Liquid | 74 |
| 18 | F-6 | 16.07.2017 |  |  | Watery | 10 |
| 19 | I-9 | 18.07.2017 |  |  | Thick | 102 |
| 20 | I-9 | 18.07.2017 |  |  | Liquid | 58 |
| 21 | L-12 | 23.07.2017 |  |  | Thick | 94 |
| 22 | I-9 | 24.07.2017 |  |  | Liquid | 44 |
| 23 | M-13 | 29.07.2017 |  |  | Watery | 46 |
| 24 | F-6 | 30.07.2017 |  |  | Thick | 98 |
| 25 | I-9 | 30.07.2017 |  |  | Liquid | 66 |
| 26 | D-4 | 16.08.2017 | 16.8 |  | Watery | 53 |
| 27 | I-9 | 07.09.2017 | 13.0 |  | Liquid | 78 |
| 28 | I-9 | 08.09.2017 |  |  | Thick | 116 |
| 29 | K-11 | 01.10.2017 | 11.0 |  | Watery | 36 |
| 30 | K-11 | 01.10.2017 |  |  | Watery | 18 |
| 31 | K-11 | 08.11.2017 | 5.3 |  | Watery | 4 |
| 32 | K-11 | 09.11.2017 |  |  | Watery | 4 |
| 33 | K-11 | 30.11.2017 |  |  | Watery | 4 |
| 34 | K-11 | 01.12.2017 | 3.2 |  | Watery | 8 |
| 35 | N-14 | 18.12.2017 |  |  | Thick | 89 |
| 36 | O-15 | 03.01.2018 | 4.5 | Winter: 2.1  Spring: 10.9  Summer: 20.0  Autumn: 10.3 | Watery | 26 |
| 37 | E-5 | 23.04.2018 | 12.8 |  | Liquid | 102 |
| 38 | I-9 | 07.06.2018 | 18.2 |  | Watery | 12 |
| 39 | P-16 | 04.09.2018 | 14.8 |  | Liquid | 78 |
| 40 | K-11 | 18.10.2018 | 10.6 |  | Watery | 45 |

* Source: [https://www.wetterkontor.de/de/wetter/deutschland/monatswerte.asp](https://www.wetterkontor.de/de/wetter/deutschland/monatswerte.asp?)
