# Supplementary material for: Quantitative assessment of German Holstein dairy cattle colostrum and impact of thermal treatment on quality of colostrum viscosity and immunoglobulins
Source: BMC Res Notes. 2020 Mar 30;13:191. doi: 10.1186/s13104-020-05019-z (PMC7106573; doi:10.1186/s13104-020-05019-z)
Supplement: Supplementary file 2 — Additional file 2: Table S2. Categorization of IgG concentrations (mg mL−1) and visual viscosity of fresh first postpartum and heat-treated colostrum samples. [file 13104_2020_5019_MOESM2_ESM.docx]

**Table S2:**

Categorization of IgG concentrations (mg mL^-1^) and visual viscosity of fresh first postpartum and heat-treated colostrum samples.

| IgG values (mg mL^-1^) | (n) | Visual viscosity of fresh first postpartum colostrum (n) | Visual viscosity of first postpartum colostrum after heat-treatment | | |
| --- | --- | --- | --- | --- | --- |
|  |  |  | 60°C/60 min (n) | 63.5°C/30 min (n) | 72°C/15 s (n) |
| 4-46 | 15 | Watery (13)  Liquid (2) | Liquid (14) | Liquid (14) | Liquid^§^ (15) |
| 50-78 | 14 | Watery (1)  Liquid (13) | Liquid (15) | Liquid^§^ (15) | Liquid^§*^ (2)  Solid^*^  (12) |
| 80-116 | 11 | Liquid (2)  Thick (9) | Thick^§^ (11) | Thick^§*^ (11) | Solid^*^ (11) |

(n) number of samples; ^§^ samples with slight (+) to moderate (++) coagulation; ^§*^ samples with moderate (+++) coagulation;

^*^ samples with excessive coagulation.
