# Supplementary material for: Quantitative assessment of German Holstein dairy cattle colostrum and impact of thermal treatment on quality of colostrum viscosity and immunoglobulins
Source: BMC Res Notes. 2020 Mar 30;13:191. doi: 10.1186/s13104-020-05019-z (PMC7106573; doi:10.1186/s13104-020-05019-z)
Supplement: Supplementary file 3 — Additional file 3: Fig. S1. Statistical correlation analysis of dairy cattle colostrum samples, Panels A, B and C: Visual viscosity with IgG (mg mL−1, %Brix and nD) concentrations. Panels D, E and F: Dynamic viscosity with IgG (mg mL−1, %Brix and nD) concentrations. Panel G: statistical correlation analysis between visual and dynamic viscosity. [file 13104_2020_5019_MOESM3_ESM.docx]

**C**

**E**

**D**

**A**

**B**

**F**

**G**

**Figure S2:**

Statistical correlation analysis of dairy cattle colostrum samples, Panels A, B and C: Visual viscosity with IgG (mg mL^-1^, %Brix and nD) concentrations. Panels D, E and F: Dynamic viscosity with IgG (mg mL^-1^, % Brixand nD) concentrations. Panel G: statistical correlation analysis between visual and dynamic viscosity.
